# Association of Phenylacetylglutamine and Cognitive Impairment in CKD

**DOI:** 10.1016/j.ekir.2025.05.037

**Published:** 2025-05-29

**Authors:** Hélène Levassort, Julie Boucquemont, Sophie Liabeuf, Solène M. Laville, Céline Lange, Luc Frimat, Christian Combe, Denis Fouque, Maurice Laville, Christian Jacquelinet, Yves-Edouard Herpe, Islam Amine Larabi, Jean-Claude Alvarez, Natalia Alencar de Pinho, Marion Pépin, Ziad A. Massy, CKD-REIN Study Collaborators, Natalia Alencar de Pinho, Dorothée Cannet, Christian Combe, Denis Fouque, Luc Frimat, Aghilès Hamroun, Yves-Edouard Herpe, Christian Jacquelinet, Maurice Laville, Sophie Liabeuf, Ziad A. Massy, Pascal Morel, Christophe Pascal, Roberto Pecoits-Filho, Joost Schanstra, Bénédicte Stengel, Céline Lange, Oriane Lambert, Marie Metzger

**Affiliations:** 1 Geriatric Department, University Medical Department 1, AP-HP, Ambroise Paré Hospital, University Hospital Group Pais Saclay, Boulogne-Billancourt, France; 2 Clinical Epidemiology Team, Centre for Research in Epidemiology and Population Health, Paris-Saclay University, Inserm U1018, Versailles Saint-Quentin University, Villejuif, France; 3 Pharmacoepidemiology Unit, Department of Clinical Pharmacology, Amiens-Picardie University Medical Center, FAmiens, France; 4 MP3CV Laboratory, Jules Verne University of Picardie, Amiens, France; 5 Nephrology Department, University Regional Hospital of Nancy, Vandoeuvre-lès-Nancy, France; 6 APEMAC, University of Lorraine, Nancy, France; 7 Bordeaux University, INSERM, Bordeaux Public Health, Bordeaux, France; 8 Hospices Civils de Lyon, Lyon, France; 9 Hospital Center of Ardèche Méridionale, Aubenas, France; 10 Service de Néphrologie, Centre Hospitalier Lyon Sud, Université de Lyon, Carmen, Pierre-Bénite, France; 11 Carmen INSERM U1060, Université Claude Bernard Lyon 1, Pierre-Bénite, France; 12 Agence de la Biomédecine, La Plaine Saint-Denis, France; 13 Biobanque de Picardie, Biological Resource Center of the Amiens University Hospital, Amiens, France; 14 Department of Pharmacology and Toxicology, Raymond Poincaré Hospital, AP-HP, Garches, France; 15 Équipe MOODS, Inserm U1018, Centre for Research in Epidemiology and Population Health, MasSpecLab, UVSQ, Université Paris-Saclay, Montigny-le-Bretonneux, France; 16 Association Pour l’Utilisation du Rein Artificiel dans la région parisienne, Paris, France; 17 Nephrology Department, University Medical Department 4, AP-HP, University Hospital Group Pais Saclay, Ambroise Paré Hospital, Boulogne-Billancourt, France

**Keywords:** chronic kidney disease, cognition, phenylacetylglutamine, uremic toxins

## Abstract

**Introduction:**

Chronic kidney disease (CKD) leads to the accumulation of uremic toxins (UTs). Studies have suggested that UTs are associated with cognitive impairment (CI) in patients with CKD. Recently, studies reported that phenylacetylglutamine (PAG) contributes to the association between CKD and CI. However, this association has not been investigated in nondialysis-dependent adults with CKD.

**Methods:**

The CKD–Renal Epidemiology and Information Network (CKD-REIN) cohort study included 3033 patients with CKD stages 2 to 5. This cross-sectional analysis included those with a PAG measurement and a mini-mental state examination (MMSE) score within 3 months of each other. CI was defined as an MMSE score ≤ 26 out of 30. Logistic regression was used to assess the association between PAG and CI.

**Results:**

Of the 2590 patients included (mean [SD] age: 67 [13] years, mean [SD] estimated glomerular filtration rate [eGFR]: 34 [13] ml/min per 1.73 m^2^, median [interquartile range, IQR] PAG level: 2.1 [1.2–3.6] mg/l), 908 (35%) presented an MMSE score ≤ 26 out of 30. After adjustment for sociodemographic factors (age, male sex, and educational level), cardiovascular risk factors, cerebrovascular disease, current depression, eGFR, urinary albumin-to-creatinine ratio (uACR), and UTs known to be associated with CI risk, a 2-fold increase in the PAG level was associated with CI (odds ratio [OR] [95% confidence interval]: 1.12 [1.01–1.23]).

**Conclusion:**

This study shows that a higher serum PAG level was associated with CI in nondialysis-dependent adults with CKD and highlight a new UT associated with CI in patients with CKD. Further studies are needed to confirm the causal nature of the association and to explore strategies for reducing serum PAG levels to protect cognition.

## Graphical abstract

**Figure:**
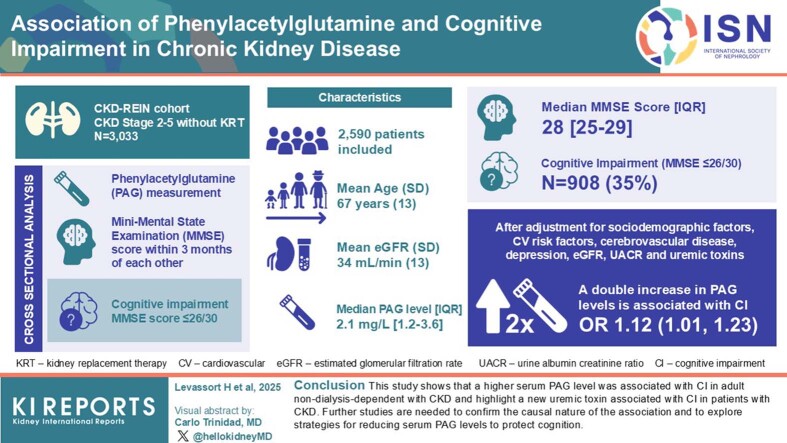

CKD affects almost 10% of the world’s population and is a major public health issue. The severity of this disease is related not only to the damage to the kidney itself but also to all the associated complications (particularly cardiovascular and neurological complications).^1^, ^2^, ^3^, ^4^ CI (ranging from mild CI to severe major neurocognitive disorder) represents a considerable burden because it develops more quickly and is more severe in patients with CKD than in the age-matched general population.^5^^,^^6^

A number of interrelated mechanisms are thought to underlie the association between CKD and CI. These include vascular and atheromatous damage caused by classical cardiovascular risk factors, the endothelial disfunction frequently encountered in CKD, and the negative effects of UTs (the production and accumulation of which are elevated in patients with CKD as a result of gut dysbiosis and poor excretion).^7^ UTs not only amplify vascular damage but also cause microscopic lesions (neuroinflammation, disruption of the blood-brain barrier, impaired neurogenesis, and perturbation of the glymphatic system) directly, leading to the onset of CI.^3^^,^^8^, ^9^, ^10^, ^11^, ^12^, ^13^

Three prominent UTs have been extensively studied in CKD: indoxyl sulfate and *p*-cresyl sulfate are both tryptophan byproducts, and trimethylamine-N-oxide (TMAO) is a byproduct of the bacterial metabolism of dietary choline and L-carnitine. According to the literature data, these 3 UTs are linked to CI.^14^, ^15^, ^16^, ^17^, ^18^, ^19^, ^20^ It was recently reported that the UT, PAG also presents vascular and neurological toxicity.^20^, ^21^, ^22^, ^23^, ^24^ PAG is a microbial metabolite of phenylalanine and is formed by the conjugation of glutamine to phenylacetic acid.^25^ Poor excretion of these 4 UTs (*p*-cresyl sulfate, indoxyl sulfate, TMAO, and PAG) in CKD leads to serum levels that are over 100 times higher in hemodialyzed patients than in healthy volunteers.^26^, ^27^, ^28^, ^29^

Although data on the association between CI and levels of *p*-cresyl sulfate, indoxyl sulfate, and TMAO are increasingly available (notably via the UTs’ effects on oxidative stress, senescence, blood-brain barrier disruption, and neuroinflammation), a role of PAG has yet to be demonstrated.^20^ A recent study of a pediatric CKD population showed an association between PAG and CI.^30^ However, to the best of our knowledge, the putative association between PAG and CI has not been investigated in a large-scale study of a cohort of nondialysis-dependent adults (i.e., not having undergone kidney replacement therapy [KRT]).

The objective of the present cross-sectional study was therefore to assess the association between PAG and CI in a large, well-characterized cohort of adult patients with CKD but no KRT at baseline. We hypothesized that independently of sociodemographic factors, cardiovascular risk factors, kidney function, and other UTs known to be associated with CI, a higher serum level of PAG is associated with poorer cognitive performance. The results of the present study might provide an additional physiological explanation for the association between CKD and CI.

## Methods

### Study Design and Population

We conducted a cross-sectional analysis of baseline data from the CKD-REIN cohort study (ClinicalTrials.gov identifier: NCT 03381950).^31^ A total of 3033 patients with CKD stage 2 to 5 were recruited from 40 randomly selected nephrology facilities in mainland France between July 2013 and April 2016. Patients had to be aged ≥ 18 years, able to provide written informed consent, and have an eGFR between 15 and 60 ml/min per 1.73 m^2^ (measured twice, at least 1 month apart) in the screening phase. Previous maintenance KRT was an exclusion criterion. Patients underwent a routine visit every year for 5 years or for up to 6 months after the initiation of KRT. The study protocol was approved by the institutional review board at the French National Institute of Health and Medical Research (*Institut National de la Santé et de la Recherche Médicale*, Paris, France; reference: IRB 00003888).

**Figure 1:**
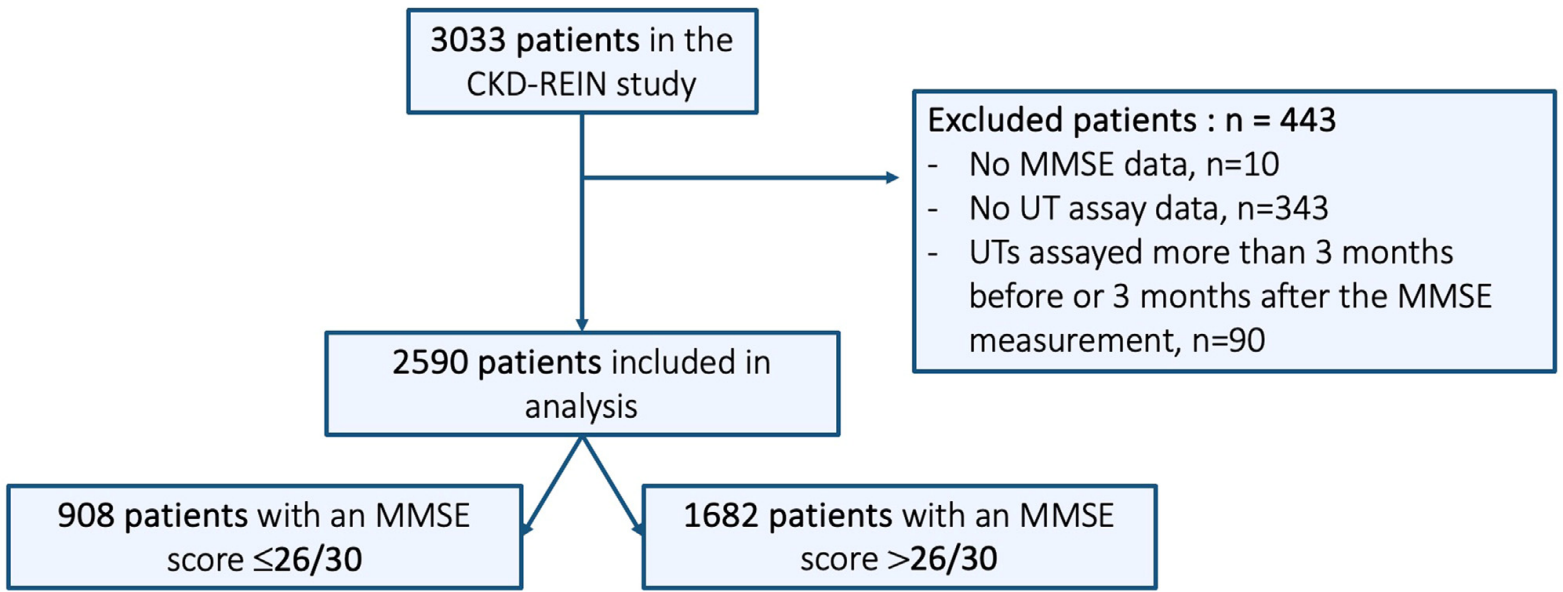
Study flowchart. MMSE, Mini-Mental State Examination; UTs, uremic toxins.

To be included in the present study, patients were required to have undergone both MMSE assessment and PAG measurement within a maximum interval of 3 months between the 2 measurements (Figure 1).

### Data

Clinical research associates collected baseline and follow-up data annually from medical records, patients interviews, and patient self-questionnaires. Patients were screened for a history of hypertension, diabetes, dyslipidemia, current smoking, cerebrovascular disease, cardiovascular disease, heart failure, and a history of cognitive disease and/or depression. The sociodemographic characteristics recorded were age, sex, educational level, and living alone or not. Symptoms of depression were assessed with the short-form Center for Epidemiologic Studies Depression scale. Current depression was defined as either the use of antidepressant medication or a Center for Epidemiologic Studies Depression scale short form score ≥ 10 of 30. This cutoff is recognized in several studies as effective for detecting depression (with a higher score indicating more pronounced depressive symptoms).^32^ Height and weight were measured, and the body mass index was calculated. A specific electronic case report form (linked to the international Anatomical Therapeutic and Chemical thesaurus) was used by the clinical research associates to record the drugs prescribed to patients in the 3 months before the enrolment visit.

Standard blood and urine tests (i.e., those recommended by the French health authorities for the routine management of CKD) were carried out for all patients at their usual medical laboratory. The GFR was estimated using the 2009 CKD–Epidemiology Collaboration creatinine equation.^18^^,^^33^ The uACR was either measured directly or estimated from proteinuria measurements.^34^^,^^35^ More details of data collection are given in Supplementary Table S1.

### Exposure: UTs Assays

All CKD-REIN samples were stored at −80 °C at the Biobanque de Picardie biological resource center (Amiens, France; BRIF number: BB-0033–00 017) and shipped frozen to Paris for analysis. Serum levels of PAG; indoxyl sulfate; *p*-cresyl sulfate; TMAO, and the PAG precursor, phenylalanine were assayed using a validated liquid chromatography - tandem mass spectrometry technique (more details are presented in Supplementary Table S1).^36^ Given that *p*-cresyl sulfate and indoxyl sulfate are mainly bound to albumin, we considered the free fraction for these 2 UTs and the total fraction for phenylalanine, TMAO, and PAG.

### Definition of CI

Trained clinical research associates assessed cognitive function with the MMSE score. This 30-item hetero-questionnaire (in which each question scores 1 point) is frequently used to screen for CI before further in-depth investigations. Patients completed the MMSE at baseline and after 5 years of follow-up. For logistic reasons, only the first 1200 patients were given the MMSE at their 2-year follow-up visit.

In this cross-sectional study, we analyzed the first available MMSE score (when multiple scores were available) that was paired with UTs (including PAG) assay data collected within a 3-month time before or after this first MMSE assessment.

Although an MMSE score < 24 is the threshold typically used to detect major neurocognitive disorders (previously known as dementia), we decided to use a threshold of ≤ 26; the latter is frequently used in epidemiologic studies to detect mild CI in patients lacking a major neurocognitive disorder.^37^^,^^38^ Furthermore, the ability to provide informed consent was a study inclusion criterion; thus, we did not expect to observe any patients with a major neurocognitive disorder or an MMSE score < 24 out of 30.

### Analyses

First, we described the baseline characteristics for the population as a whole (*N* = 2590) and with regard to tertiles of the baseline serum PAG level (< 1.43 mg/l, 1.43–2.973 mg/l, and > 2.973 mg/l) (Table 1). An analysis of variance or the Kruskal-Wallis test was applied to continuous variables (depending on the normality of distribution), and a chi-square test was applied to categorical variables.

**Table 1:** Baseline characteristics of the participants (*N* = 2590), according to the total PAG level

Characteristics	Missing data, n,(%)	Overall *n* = 2590	PAG level			*P*^a^
			Low tertile (T1) *n* = 865 < 1.43 mg/l	Medium tertile (T2) *n* = 863 1.43–2.973 mg/l	High tertile (T3) *n* = 862 > 2.973 mg/l	
Sociodemographic factors						
Age, yr, mean (SD)		66.5 (12.9)	62.6 (13.2)	67.7 (12.2)	69.3 (12.2)	< 0.001
Males (%)	0	1710 (66.0)	576 (66.6)	552 (64.0)	582 (67.5)	0.3
Educational level≥ 12 yrs, (%)	24 (0.9)	929 (35.9)	326 (37.7)	313 (36.3)	290 (33.6)	0.4
Living alone (%)	360 (13.9)	503 (19.4)	165 (19.1)	169 (19.6)	169 (19.6)	0.7
Baseline drug prescriptions						
Polymedication (≥ 5/d) (%)	8 (0.3)	2075 (80.1)	611 (70.6)	691 (80.1)	773 (89.7)	< 0.001
Antidepressants (%)	8 (0.3)	193 (7.5)	47 (5.4)	66 (7.6)	80 (9.3)	0.01
Anxiolytics (%)	8 (0.3)	275 (10.6)	74 (8.6)	92 (10.7)	109 (12.6)	0.02
Psychoactive drugs^b^(%)	8 (0.3)	514 (19.8)	134 (15.5)	169 (19.6)	211 (24.5)	< 0.001
Cardiovascular risk factors^c^						
Hypertension, %	0	2333 (90.1)	759 (87.7)	780 (90.4)	794 (92.1)	0.009
Diabetes mellitus, %	0	1087 (42.0)	310 (35.8)	347 (40.2)	430 (49.9)	< 0.001
Dyslipidemia, %	0	1892 (73.1)	613 (70.9)	615 (71.3)	664 (77.0)	0.005
BMI, mean (SD)	33 (1.5)	28.72 (5.9)	28.69 (5.9)	28.71 (5.7)	28.77 (6.0)	0.9
Current smoking, %	127 (4.9)	298 (11.5)	125 (14.5)	93 (10.8)	80 (9.3)	0.01
Comorbidities						
Cerebrovascular disease^d^ (%)	0	295 (11.4)	77 (8.9)	87 (10.1)	131 (15.2)	< 0.001
Heart failure^e^ (%)	4 (0.2)	328 (12.7)	70 (8.1)	108 (12.5)	150 (17.4)	< 0.001
Cardiovascular disease^f^ (%)	12 (0.5)	1340 (51.7)	368 (42.5)	438 (50.8)	534 (61.9)	< 0.001
Short-form CESD score (/30), median [IQR]	476 (18.4)	7.0 [4.0, 10.0]	6.0 [3.0, 10,0]	7.0 [4.0, 10.0]	7 [4.0, 11.0]	< 0.001
Current depression^g^, (%)	446 (17.2)	724 (28.0)	493 (57.0)	475 (55.0)	452 (52.4)	0.01
History of cognitive disease (%)	16 (0.6)	15 (0.6)	3 (0.3)	3 (0.3)	9 (1.0)	0.07
MMSE total score (/30), median [IQR]	0	28 [25–29]	28 [26–29]	28 [25–29]	27 [25–29]	< 0.001
Standard laboratory variables						
eGFR (ml/min per 1.73 m^2^), mean (SD)	4 (0.1)	34.2 (13.3)	41.3 (13.3)	34.7 (11.8)	26.8 (10.6)	< 0.001
uACR, mean (SD)	411 (15.9)					< 0.001
Normal: < 3 mg/mmol		636 (24.6)	272 (31.4)	224 (26.0)	140 (16.2)	
Moderate elevation: 3–30 mg/mmol		752 (29.0)	205 (23.7)	230 (26.7)	317 (36.8)	
Severe elevation: > 30 mg/mmol		791 (30.5)	258 (29.8)	273 (31.6)	260 (30.2)	
Uremic toxins						
Serum total phenylalanine level (mg/l), median [IQR]	0	16.8 [14.3–20.1]	16.5 [13.8–19.0]	16.5 [14.2–19.8]	18.1 [15.1–21.7]	< 0.001
Serum total PAG level (mg/l), median [IQR]	0	2.09 [1.2–3.6]	0.9 [0.6–1.2]	2.1 [1.8–2.5]	4.5 [3.6–6.2]	< 0.001
Serum total TMAO level (mg/l), median [IQR]	0	1.3 [0.7–2.3]	0.8 [0.5–1.4]	1.2 [0.7–1.9]	2.0 [1.2–3.5]	< 0.001
Serum free indoxyl sulfate level (mg/l), median [IQR]	0	0.05 [0.03–0.09]	0.03 [0.02–0.05]	0.05 [0.03–0.08]	0.1 [0.06–0.2]	< 0.001
Serum free p-cresyl sulfate level (mg/l), median [IQR]	0	0.2 [0.09–0.3]	0.07 [0.04–0.1]	0.2 [0.1–0.3]	0.4 [0.3–0.7]	< 0.001

BMI, body mass index; CESD, Center for Epidemiologic Studies Depression; eGFR, estimated glomerular filtration rate; IQR, interval interquartile; MMSE, Mini Mental State Examination; PAG, phenylacetylglutamine; TMAO, trimethylamine oxide; uACR, urinary albumin-to-creatinine ratio.

a ANOVA for comparisons of means, chi-squared test for comparisons of percentages, and the Kruskal-Wallis test for comparisons of category distributions.

b Psychoactive drug intake was defined as taking at least 1 antidepressant, anxiolytic, or antipsychotic.

c Hypertension was defined as a history of hypertension or the use of blood-pressure–lowering medication. Diabetes mellitus was defined as a history of diabetes, antidiabetic medication use, a glycosylated hemoglobin level ≥ 6.5%, a fasting glycemia value ≥ 7 mmol/l, or a nonfasting glycemia value ≥ 11 mmol/l. Dyslipidemia was defined as a history of dyslipidemia or the use of lipid-lowering medication. Obesity was defined as a body mass index ≥ 30 kg/m^2^. Current smoking was defined as at least 1 cigarette/d.

d Cerebrovascular disease was defined as a history of stroke, transient ischemic attack, or brain hemorrhage.

e Heart failure was defined as an history of heart failure or pulmonary edema.

f History of coronary heart disease, angina pectoris, myocardial infarction, coronary bypass surgery, percutaneous coronary intervention, cardiac arrest, atrial fibrillation, other heart rhythm disorder, an implanted pacemaker, implanted defibrillator, heart failure, pulmonary edema, pericarditis, heart valve disease, heart valve prosthesis, stroke, transient ischemic attack, endarterectomy, cerebral hemorrhage, peripheral vascular disease, intermittent claudication, arterial bypass/percutaneous intervention for arteritis, renal artery stenosis/renal artery surgery, or aortic aneurysm, or surgical treatment of an aortic aneurysm.

g Current depression was described as a CESD short form ≥ 10 of 30 or antidepressant intake.

In the main analysis, logistic regression was used to assess the association between the serum PAG level and an MMSE score ≤ 26 out of 30. Given that the serum PAG values were not normally distributed, we log-transformed the data (log2[PAG]). Therefore, the resulting OR for an MMSE score ≤ 26 out of 30 relates to a 2-fold increase in the PAG level. The linear functional form was used because it gave a better fit than a smoother form with a restricted cubic spline (Akaike information criterion, or Bayesian information criterion x and y, respectively). The selection of the following variables included in the model was based on a review of the literature: age, male sex, educational level, hypertension, diabetes, dyslipidemia, body mass index (per 5 kg/m^2^ increment), current smoking, cerebrovascular disease, current depression, eGFR (per 10 ml/min per 1.73 m^2^ decrement), uACR, and UTs known to be associated with cognitive function (TMAO, indoxyl sulfate, and *p*-cresyl sulfate). The models were adjusted progressively as follows: model 1 was adjusted for the nonmodifiable sociodemographic factors; model 2 additionally included cardiovascular risk factors, cerebrovascular disease, current depression, and the 3 UTs; and model 3 additionally included the kidney function variables eGFR and uACR. Given the limited knowledge regarding interactions between UTs, and in order to capture the overall burden of UTs in patients with CKD (and thus to study how much adding the estimation of serum PAG concentration increases the risk of developing CI in this population) we created a variable representing the sum of TMAO, free indoxyl sulfate, and free p-cresyl sulfate. This approach has been used in other studies investigating the effects of toxic compounds.^39^ This variable was included in models 2 and 3. Given that the serum level of all UTs increases with the CKD stage, we tested for interactions between the sum of UTs and the eGFR. Moreover, we tested for collinearity between variables by calculating the variance inflation factor (in the *car* package in R). Analyses investigating the association between levels of free indoxyl sulfate, total TMAO, and free p-cresyl sulfate were also conducted and served as control analyses.

In a sensitivity analysis, PAG tertiles were considered as a categorical variable, with the lowest tertile as the reference. The same adjustments were applied. In another sensitivity analysis, we studied the association between the serum PAG level and the MMSE threshold known to detect major neurocognitive disorders with the greatest sensitivity and specificity (MMSE < 24/30; sensitivity: 0.85; specificity: 0.90).^38^

Multiple imputation of missing data with chained equations was applied, with the creation of 30 datasets of 30 iterations each.^40^ All variables presented in the logistic regressions were included in the imputation procedure. ORs were quoted with their corresponding 95% confidence interval, and the threshold for statistical significance was set to *P* < 0.05. All statistical analyses were performed with R software (version 2024.04.2 R Foundation for Statistical Computing, Vienna, Austria).^41^

## Results

### Characteristics of the Study Population

After the application of our exclusion criteria, 2590 patients were included in the analysis of PAG tertiles (Table 1 and Figure 1). The mean (SD) age was 67 (13) years and the mean (SD) eGFR was 34 (13) ml/min per 1.73 m^2^. The median [IQR] serum PAG level was 2.1 [1.2–3.6] mg/l. We observed significant differences in the eGFR and the uACR between the 3 groups. The mean eGFR for patients in the highest PAG tertile was 27 ml/min per 1.73 m^2^, and 67% of these patients had a uACR ≥ 3 mg/mmol. The median [IQR] MMSE score was 28 [25–29], and 908 of the 2590 patients (35%) had an MMSE score ≤ 26 out of 30. The MMSE score was significantly lower for the patients in the middle and highest PAG tertiles (*P* < 0.001). Patients with a higher serum PAG level were older and more likely to have diabetes mellitus (*P* < 0.001), hypertension (*P* = 0.009), cerebrovascular disease (*P* < 0.001), and polymedication (*P* < 0.001). The phenylalanine concentration increased gradually from the lowest to the highest PAG tertile (Table 1).

The serum PAG level increases with the CKD stage: the median [IQR] value was 0.8 [0.5–1.3] mg/l for patients with CKD stage 2 and 5.6 [3.4–8.0] mg/l for patients with CKD stage 5 (Supplementary Figure S1A). The serum phenylalanine level did not vary greatly as a function of the CKD stage with a median [IQR] value of 15.19 [13.2–18.6] mg/l in patients with CKD stage 2 and 14.6 [12.4–17.0] mg/l in patients with CKD stage 5 (Supplementary Figure S1B).

### The Association Between Serum PAG and an MMSE Score ≤ 26 out of 30

**Figure 2:**
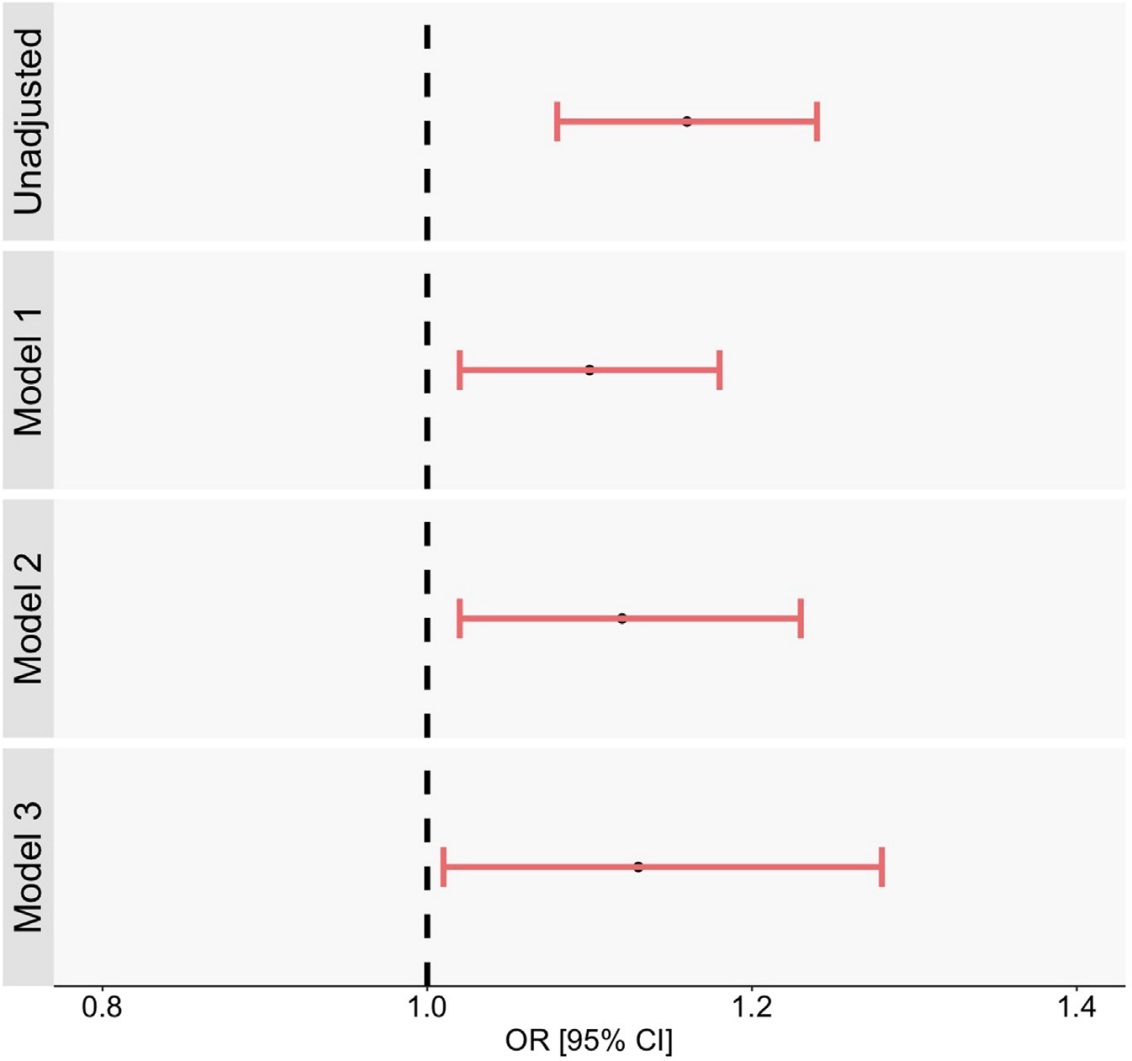
Association between log_2_[total PAG] and a MMSE score ≤ 26/30, in unadjusted and adjusted logistic regressions (*N* = 2590). Model 1 was adjusted for log_2_[total PAG], age, male sex, and educational level. Model 2 was adjusted for log2[total PAG], age, male sex, educational level, hypertension, diabetes mellitus, dyslipidemia, obesity, current smoking, cerebrovascular disease, depression, log_2_Σ(total TMAO, free IS, free pCS). Model 3 was adjusted for Log2 [total PAG], age, male sex, educational level, hypertension, diabetes mellitus, dyslipidemia, obesity, current smoking, cerebrovascular disease, depression, log2S(total TMAO, free IS, free pCS), eGFR and uACR. CI, confidence interval; eGFR, estimated glomerular filtration rate oxide; IS, indoxyl sulfate; MMSE, Mini-Mental State Examination; OR, odds ratio; PAG, phenylacetylglutamine; pCS, p-cresyl sulfate; TMAO, trimethylamine; uACR, urinary albumin-to-creatinine ratio.

The crude analysis showed that a 2-fold increase in the serum PAG level was associated with a significantly greater likelihood of an MMSE score ≤ 26 out of 30 (OR [95% confidence interval]: 1.16 [1.08–1.24]) (Figure 2, Table 2). After multiple adjustments for sociodemographic and clinical factors, this association was still significant. In model 3, a 2-fold increase in the serum PAG level was associated with a 15% greater likelihood of an MMSE score ≤ 26 out of 30 [adjusted OR (95% confidence interval): 1.12 (1.01–1.23)].

**Table 2:** Association between log2 [total PAG] and a MMSE score **≤** 26 out of 30 in an adjusted logistic regression (*N* = 2590)

	Unadjusted		Model 1		Model 2		Model 3	
	OR [95% CI]	*P*	OR [95% CI]	*P*	OR [95% CI]	*P*	OR [95% CI]	*P*
Uremic toxins								
Log2 [total PAG]	1.16 [1.08–1.24]	< 0.001	1.10 [1.02–1.18]	0.009	1.12 [1.02–1.23]	0.01	1.12 [1.01–1.23]	0.03

CI, confidence interval; eGFR, estimated glomerular filtration rate; IS, indoxyl sulfate; Log2, based-2 logarithm; MMSE, Mini Mental State Examination; OR, odds ratio; PAG, phenylacetylglutamine; pCS, p-cresyl sulfate; TMAO, trimethylamine oxide; uACR, urinary albumin-to-creatinine ratio.

Model 1 was adjusted for Log2 [total PAG], age, male sex, and educational level.

Model 2 was adjusted for Log2 [total PAG], age, male sex, educational level, hypertension, diabetes mellitus, dyslipidemia, obesity, current smoking, cerebrovascular disease, depression, log2S(total TMAO, free IS, free pCS).

Model 3 was adjusted for Log2 [total PAG], age, male sex, educational level, hypertension, diabetes mellitus, dyslipidemia, obesity, current smoking, cerebrovascular disease, depression, log2S(total TMAO, free IS, free pCS), eGFR, and uACR.

A doubling of the free indoxyl sulfate concentration was associated with MMSE ≤ 26 out of 30, (OR [95% confidence interval]: 1.10 [1.01–1.19]) (Supplementary Table S2).

### Sensitivity Analyses

In the first sensitive analysis, the highest PAG tertile was associated with a significantly greater likelihood of an MMSE score ≤ 26 out of 30 (relative to the lowest tertile). The adjusted ORs (95% confidence interval) were 1.36 (1.09–1.68), 1.43 (1.11–1.85), and 1.41 (1.09–1.84) for models 1, 2, and 3, respectively (Table 3). There were no interactions or collinearities between the sum of UTs and the eGFR.

**Table 3:** Association between [total PAG] (considered in tertiles) with the MMSE score ≤ 26/30 in an adjusted logistic regression (***n*** = 2590)

	Unadjusted		Model 1		Model 2		Model 3	
	OR [95% CI]	*P*	OR [95% CI]	*P*	OR [95% CI]	*P*	OR [95% CI]	*P*
Uremic toxins								
PAG								
∗ 1^st^ tertile	Reference		Reference		Reference		Reference	
∗ 2^nd^ tertile	1.16 (0.95–1.42)	0.2	1.03 (0.83–1.28)	0.8	1.06 (0.85–1.33)	0.6	1.06 (0.84–1.33)	0.7
∗ 3^rd^ tertile	1.56 (1.26–1.90)	<0.001	1.36 (1.09–1.68)	0.005	1.43 (1.11–1.85)	0.006	1.41 (1.09–1.84)	0.01

CI, confidence interval; eGFR, estimated glomerular filtration rate; Log2, based-2 logarithm; IS, indoxyl sulfate; MMSE, Mini Mental State Examination; OR, odds ratio; PAG, phenylacetylglutamine; pCS, p-cresyl sulfate; TMAO, trimethylamine oxide; uACR, urinary albumin-to-creatinine ratio.

Model 1 was adjusted for Log2 [total PAG], age, male sex, and educational level.

Model 2 was adjusted for Log2 [total PAG], age, male sex, educational level, hypertension, diabetes mellitus, dyslipidemia, obesity, current smoking, cerebrovascular disease, depression, log2S(total TMAO, free IS, free pCS).

Model 3 was adjusted for Log2 [total PAG], age, male sex, educational level, hypertension, diabetes mellitus, dyslipidemia, obesity, current smoking, cerebrovascular disease, depression, log2S(total TMAO, free IS, free pCS), eGFR and uACR.

In the second sensitivity analysis, we applied an MMSE threshold of < 24 out of 30: 323 of the 2590 patients (12.5%) presented an MMSE score below this value. After adjustment for all the covariates, the effect size was associated with a 2-fold increase in the serum PAG level (OR [95% confidence interval]: 1.05 [0.91–1.20], Supplementary Table S3). Regarding indoxyl sulfate, we did not find any association between a doubling of this UT value and an MMSE score < 24 out of 30 (Supplementary Table S4).

## Discussion

In 2 analyses of the association between the serum PAG concentration and CI in a large cohort of adults with CKD but not KRT, we observed a constant association between higher serum PAG levels and CI (defined as an MMSE score ≤ 26 out of 30). The main analysis showed that a 2-fold increase in the serum PAG level was associated with CI, and the sensitivity analysis showed that patients with higher serum PAG levels (based on the population’s tertile distribution) had a greater likelihood of presenting CI than patients with lower levels. The results of these analyses suggest that PAG has a specific role in CKD-related CI, independently of age, sex, educational level, cardiovascular risk factors, cerebrovascular disease, current depression, eGFR, uACR, and other UTs known to increase the incidence of CI (indoxyl sulfate, *p*-cresyl sulfate, and TMAO). Our results also confirm the association between indoxyl sulfate levels and MMSE score, which is consistent with numerous studies.^9^^,^^14^^,^^15^ This study constitutes a strong rationale for studies evaluating PAG’s toxicity in adults with pre-KRT CKD because previously published studies had small sample sizes or included dialysis patients only.^23^

As interest in the kidney-brain axis grows, the research community is focusing on intestinal microbial metabolism as a factor that contributes to the accumulation of solutes present in CKD; the main emphasis is on TMAO, PAG, indoxyl sulfate, and *p*-cresyl sulfate.^7^^,^^20^ TMAO, indoxyl sulfate and *p*-cresyl sulfate have been linked to a cardiovascular risk with endothelial damage through inflammation and collagen deposition, which both contribute to the development of atherosclerotic plaques, arterial calcification, and arterial stiffness.^42^, ^43^, ^44^ A different metabolic pathway for PAG might explain its vascular involvement, for example via the α2A, α2B, and β2 adrenergic receptors that are strongly expressed on platelets and have been linked to platelet activation and thrombosis.^25^^,^^45^

Our results showed that PAG was associated with an increased risk of CI, independently of cardiovascular risk factors, cerebrovascular disease, current depression, and other UTs known to promote CI; this finding suggests that PAG has specific neurotoxicity, in addition to its vascular toxicity. A specific association between PAG and CI has been found in the following: (i) preclinical studies of mouse models; (ii) epidemiological studies of pediatric patients with CKD, patients on hemodialysis, and patients with Alzheimer’s disease and (iii) studies of PAG levels and structural brain damage.^23^^,^^30^^,^^46^, ^47^, ^48^ In particular, a study of patients with multiple sclerosis showed an inverse correlation between PAG, *p*-cresyl sulfate, and indoxyl sulfate levels and MRI measurements of the cortical volume; the latter is directly correlated with the level of neurofilament light chain in the cerebrospinal fluid and thus serves as a well-established biomarker of neurodegeneration.^48^ However, it remains to be determined whether PAG’s neurotoxicity is because of a specific metabolic pathway or whether its action is similar to that of indoxyl sulfate. Indeed, indoxyl sulfate is known to inhibit glycolysis in astrocytes, which leads to cell death by apoptosis. Indoxyl sulfate is also known to damage the blood-brain barrier by activating the aryl hydrocarbon receptor.^9^^,^^16^ Furthermore, it is not yet known whether PAG has a specific role in impairments of the glymphatic system and brain neurotransmitters (disorders that are frequently described as being associated with UTs).^3^^,^^8^ PAG probably causes brain damage both directly (through a neurotoxic effect on brain tissue) and indirectly (often through subclinical cerebrovascular damage). However, there are many unknowns with regard to the neurotoxicity of PAG and other UTs. Do the UTs interact? Do they potentiate or inhibit each other? In the present study, we did not observe any interactions between the various UTs.

Our results show that the serum PAG level rises with the CKD stage. This is in line with a study in the general population, which found a median [IQR] serum PAG concentration of 0.2 [0.02–0.34] mg/l.^49^ In our study, the concentration of the PAG precursor, phenylalanine remained quite stable as the PAG level increased; this finding might indicate that an increase in phenylalanine concentration led to an increase in PAG production. Furthermore, failure to eliminate PAG (as a result of kidney damage) might contribute to the increase in PAG concentration in CKD population.^21^^,^^26^ However, our results show that independently of kidney function, the serum PAG level is associated with an elevated likelihood of CI. Further studies are needed to better understand the mechanisms by which an increase in the PAG level occurs in patients with CKD and to determine appropriate targets for reducing the compound’s toxicity. One could consider strategies for reducing its production (through dietary means or probiotics) and/or promoting its elimination (e.g., using chelators).^20^

As explained in the Methods section, we and others considered that an MMSE threshold of 26 out of 30 was more appropriate for a study population with a good cognitive level.^4^^,^^37^^,^^50^ Even though we found an association between PAG levels and an MMSE ≤ 26 out of 30, the OR value in model 3 may appear weak. However, it should be interpreted in the context of other risk factors (many of which are categorical variables) known to be strongly associated with CI. In addition, in our cohort, cognitive function was assessed using the MMSE, which is less sensitive to assess executive dysfunction, the most affected in patients with CKD. Although the MMSE is widely used and recognized worldwide, it may have underestimated the prevalence of CI in our population. The results of our sensitivity study with a threshold of < 24 out of 30 were not statistically significant, probably for several reasons as follows: the smaller number of patients with an MMSE score < 24 out of 30 reduced the statistical power, and/or the possible lack of an effect of PAG on advanced CI (i.e., a threshold effect). The lack of association between indoxyl sulfate and an MMSE score < 24 out of 30 (despite it being the most widely recognized UT associated with cognitive function) further supports our hypothesis of insufficient statistical power.

Our study has some limitations. First, the relatively small number of patients with repeated MMSE measurements prevented us from considering a longitudinal analysis. This difficulty was because of the selection bias inherent in all cohort studies, given that patients with CI adhere less well to follow-up procedures. This also resulted in a cognitively homogeneous population with overall preserved cognitive performance, which likely contributed to the weak association observed between PAG and MMSE score. Moreover, the COVID-19 pandemic had a strong impact on our collection of data and prevented a large number of patients from attending the final cognitive assessment at 5 years. Second, as explained earlier, the MMSE score was the only cognitive assessment test available in the CKD-REIN cohort, which is less sensitive to detect executive dysfunction, although the MMSE score appears to be impaired early in the course of CKD.^51^ The use of the MMSE alone probably led to a lack of sensitivity for CI screening in our cohort. Furthermore, the 30-item MMSE gives a cumulative score of 30, with 1 point per item; the MMSE score is therefore probably more akin to a discrete variable, rather than a continuous variable. This prevented us from using linear models, and we therefore opted for logistic models by dichotomizing the MMSE score as being above or below or equal to the 26 out of 30 threshold. Third, the CKD-REIN study did not collect data on the diet, and so we were unable to take this factor into account in our analysis. Lastly, our study resembled a scoping study that explored new ways of understanding the physiological mechanism(s) that underlying the association between the PAG level and cognition. It is important to note that several factors and several UTs appear to be involved in CKD-related CI, and so PAG is probably only 1 link in the chain. Further studies are needed to explore the putative causal nature of the link between PAG and CI.

Our study has many strengths. First, the datasets from the CKD-REIN cohort enabled us to assess a broad panel of variables and to adjust our models for a large number of potential confounding factors. Even after excluding individuals with a time interval between the MMSE and the UT assay of > 3 months, we were still able to include 2590 patients in our study. Second, cognitive function was evaluated using the MMSE by trained clinical research associates. Third, the UT assays were centralized and were based on a validated, robust, ultra–high-performance liquid chromatography–tandem mass spectrometry technique. Fourth, our results were robust with regard to the type of exposure: serum PAG as a continuous or categorical variable.

## Conclusion

In this study of a large cohort of adult patients with CKD, we observed an independent association between the serum PAG level and CI after multiple adjustments for sociodemographic factors, cardiovascular risk factors, cerebrovascular disease, kidney function, and other UTs known to promote CI. Our results constitute a strong rationale for further studies of adults with CKD but not KRT and contribute to a better understanding of the role of UTs in CI. Future research should determine whether UTs (including PAG) are predictive biomarkers for mild CI. Furthermore, preclinical studies of the metabolic pathways involving PAG are required, with a view to fostering the development of potential therapeutic approaches that target PAG and other UTs in the management of cognitive disorders.

## Appendix

### List of the CKD-REIN Study Collaborators

Natalia Alencar de Pinho, Dorothée Cannet, Christian Combe, Denis Fouque, Luc Frimat, Aghilès Hamroun, Yves-Edouard Herpe, Christian Jacquelinet, Maurice Laville, Sophie Liabeuf, Ziad A. Massy, Pascal Morel, Christophe Pascal, Roberto Pecoits-Filho, Joost Schanstra, Bénédicte Stengel, Céline Lange, Oriane Lambert, and Marie Metzger.

## Disclosure

ZAM reports grants for CKD-REIN and other research projects from Amgen, Baxter, Fresenius Medical Care, GlaxoSmithKline, Merck Sharp, and Dohme-Chibret, Sanofi-Genzyme, Lilly, Otsuka, Boehringer, and the French government; as well as fees and grants to charities from Astra Zeneca, GSK, and Boehringer; these sources of funding are not necessarily related to the content of the present manuscript. NAP declares financial support from pharmaceutical companies who are members of the CKD-REIN public-private partnership in the last 36 months: GlaxoSmithKline (GSK), Boehringer Ingelheim France, Novo Nordisk; all grants have been made to Paris Saclay University. CC reports honoraria from Fresenius, Astellas, and Sanofi. All the other authors declared no competing interests.

## Supplementary Material

Supplementary File (PDF) **Supplementary Methods 1.** List of biological resources centers. **Figure S1.** (A) PAG and (B) phenylalanine concentrations, by CKD stage (*N* = 2590). **Table S1.** Definitions of the operational variables used in the study. **Table S2.** Association between (A) log2[IS], (B) log2 [TMAO], (C) log2 [pCS], and an MMSE score ≤ 26/30, in unadjusted and adjusted logistic regressions (*N* = 2590). **Table S3.** Association between log2[PAG] and an MMSE score < 24/30, in unadjusted and adjusted logistic regressions (*N* = 2590). **Table S4.** Association between (A) log2[IS], (B) log2 [TMAO], (C) log2 [pCS], and an MMSE score < 24/30, in unadjusted and adjusted logistic regressions (*N* = 2590). STROBE checklist.

## Footnotes

Supplementary File (PDF)
**Supplementary Methods 1.** List of biological resources centers. **Figure S1.** (A) PAG and (B) phenylalanine concentrations, by CKD stage (*N* = 2590). **Table S1.** Definitions of the operational variables used in the study. **Table S2.** Association between (A) log2[IS], (B) log2 [TMAO], (C) log2 [pCS], and an MMSE score ≤ 26/30, in unadjusted and adjusted logistic regressions (*N* = 2590). **Table S3.** Association between log2[PAG] and an MMSE score < 24/30, in unadjusted and adjusted logistic regressions (*N* = 2590). **Table S4.** Association between (A) log2[IS], (B) log2 [TMAO], (C) log2 [pCS], and an MMSE score < 24/30, in unadjusted and adjusted logistic regressions (*N* = 2590).
**STROBE checklist.**

## References

[1] Kovesdy C.P. (2022). Epidemiology of chronic kidney disease: an update 2022. Kidney Int Suppl (2011).

[2] Jankowski J., Floege J., Fliser D., Böhm M., Marx N. (2021). Cardiovascular disease in chronic kidney disease: pathophysiological insights and therapeutic options. Circulation.

[3] Pépin M., Levassort H., Massy Z.A. (2024). The impact of chronic kidney disease on cognitive function. Curr Opin Nephrol Hypertens.

[4] Arafa A., Kawachi H., Matsumoto C. (2024). The association between the estimated glomerular filtration rate and cognitive impairment: the Suita Study. Hypertens Res Off J Jpn Soc Hypertens.

[5] Pepin M., Ferreira A.C., Arici M. (2021). Cognitive disorders in patients with chronic kidney disease: specificities of clinical assessment. Nephrol Dial Transplant.

[6] Pépin M., Levassort H., Boucquemont J. (2022). Cognitive performance is associated with glomerular filtration rate in patients with chronic kidney disease: results from the CKD-REIN cohort. J Neurol Neurosurg Psychiatry.

[7] Yang T., Richards E.M., Pepine C.J., Raizada M.K. (2018). The gut microbiota and the brain-gut-kidney axis in hypertension and chronic kidney disease. Nat Rev Nephrol.

[8] Viggiano D., Wagner C.A., Martino G. (2020). Mechanisms of cognitive dysfunction in CKD. Nat Rev Nephrol.

[9] Bobot M., Guedj E., Resseguier N. (2024). Increased blood-brain barrier permeability and cognitive impairment in patients with ESKD. Kidney Int Rep.

[10] Kelly D.M., Pinheiro A.A., Koini M. (2024). Impaired kidney function, cerebral small vessel disease and cognitive disorders: the Framingham Heart Study. Nephrol Dial Transplant Off Publ Eur Dial Transpl Assoc Eur Ren Assoc.

[11] Liabeuf S., Pepin M., Franssen C.F.M. (2021). Chronic kidney disease and neurological disorders: are uraemic toxins the missing piece of the puzzle?. Nephrol Dial Transplant.

[12] Assem M., Lando M., Grissi M. (2018). The impact of uremic toxins on cerebrovascular and cognitive disorders. Toxins.

[13] Xu S., Wang J., Sun K. (2023). Cognitive impairment in chronic kidney disease is associated with glymphatic system dysfunction. Kidney Dis Basel Switz.

[14] Jeong S.H., Park S., Choi J.S., Cho N.J., Moon J.S., Gil H.W. (2023). Indoxyl sulfate induces apoptotic cell death by inhibiting glycolysis in human astrocytes. Kidney Res Clin Pract.

[15] Yeh Y.C., Huang M.F., Liang S.S. (2016). Indoxyl sulfate, not p-cresyl sulfate, is associated with cognitive impairment in early-stage chronic kidney disease. NeuroToxicology.

[16] Salminen A. (2023). Activation of aryl hydrocarbon receptor (AhR) in Alzheimer’s disease: role of tryptophan metabolites generated by gut host-microbiota. J Mol Med Berl Ger.

[17] Brunt V.E., LaRocca T.J., Bazzoni A.E. (2021). The gut microbiome-derived metabolite trimethylamine N-oxide modulates neuroinflammation and cognitive function with aging. GeroScience.

[18] Chen X., Gu M., Hong Y., Duan R., Zhou J. (2022). Association of trimethylamine N-oxide with normal aging and neurocognitive disorders: a narrative review. Brain Sci.

[19] Yaqub A., Vojinovic D., Vernooij M.W. (2024). Plasma trimethylamine N-oxide (TMAO): associations with cognition, neuroimaging, and dementia. Alzheimers Res Ther.

[20] Schwarz A., Hernandez L., Arefin S. (2024). Sweet, bloody consumption – what we eat and how it affects vascular ageing, the BBB and kidney health in CKD. Gut Microbes.

[21] Poesen R., Claes K., Evenepoel P. (2016). Microbiota-derived phenylacetylglutamine associates with overall mortality and cardiovascular disease in patients with CKD. J Am Soc Nephrol.

[22] Song Y., Wei H., Zhou Z. (2024). Gut microbiota-dependent phenylacetylglutamine in cardiovascular disease: current knowledge and new insights. Front Med.

[23] Kurella Tamura M., Chertow G.M., Depner T.A. (2016). Metabolic profiling of impaired cognitive function in patients receiving dialysis. J Am Soc Nephrol.

[24] Rosell-Díaz M., Santos-González E., Motger-Albertí A. (2023). Gut microbiota links to serum ferritin and cognition. Gut Microbes.

[25] Saha P.P., Gogonea V., Sweet W. (2024). Gut microbe-generated phenylacetylglutamine is an endogenous allosteric modulator of β2-adrenergic receptors. Nat Commun.

[26] Sirich T.L., Aronov P.A., Plummer N.S., Hostetter T.H., Meyer T.W. (2013). Numerous protein-bound solutes are cleared by the kidney with high efficiency. Kidney Int.

[27] Sirich T.L., Funk B.A., Plummer N.S., Hostetter T.H., Meyer T.W. (2014). Prominent accumulation in hemodialysis patients of solutes normally cleared by tubular secretion. J Am Soc Nephrol.

[28] Hai X., Landeras V., Dobre M.A., DeOreo P., Meyer T.W., Hostetter T.H. (2015). Mechanism of prominent trimethylamine oxide (TMAO) accumulation in hemodialysis patients. PLOS One.

[29] Mair R.D., Lee S., Plummer N.S., Sirich T.L., Meyer T.W. (2021). Impaired tubular secretion of organic solutes in advanced chronic kidney disease. J Am Soc Nephrol.

[30] Lee A.M., Xu Y., Hooper S.R. (2024). Circulating metabolomic associations with neurocognitive outcomes in pediatric CKD. Clin J Am Soc Nephrol.

[31] Stengel B., Combe C., Jacquelinet C. (2014). The French chronic kidney disease-renal epidemiology and information network (CKD-REIN) cohort study. Nephrol Dial Transplant.

[32] Center for Epidemiologic Studies Depression Scale. Shortened Version.pdf. https://alswh.org.au/wp-content/uploads/2020/08/DDSSection2.7CESD.pdf.

[33] Levey A.S., Stevens L.A., Schmid C.H. (2009). A new equation to estimate glomerular filtration rate. Ann Intern Med.

[34] Sumida K., Nadkarni G.N., Grams M.E. (2020). Conversion of urine protein–creatinine ratio or urine dipstick protein to urine albumin–creatinine ratio for use in chronic kidney disease screening and prognosis. Ann Intern Med.

[35] Levin A., Stevens P.E., Bilous R.W. (2013). Kidney disease: improving global outcomes (KDIGO) CKD work group. KDIGO 2012 clinical practice guideline for the evaluation and management of chronic kidney disease. Kidney Int Suppl.

[36] Aregui A., Qabbal R., Culine S., Diaz V.F. (2019). [The quality and quantity of life of the elderly person in oncology]. Soins Gerontol.

[37] Arevalo-Rodriguez I., Smailagic N., Roqué-Figuls M. (2021). *Mini-Mental State Examination (MMSE) for the Early Detection of Dementia in People With Mild Cognitive Impairment (MCI) - Arevalo-Rodriguez*, I: Cochrane Library. https://www.cochranelibrary.com/cdsr/doi/10.1002/14651858.CD010783.pub3/full.

[38] Creavin S.T., Wisniewski S., Noel-Storr A.H. (2016). Mini-mental state examination (MMSE) for the detection of dementia in clinically unevaluated people aged 65 and over in community and primary care populations. Cochrane Database Syst Rev Cochrane.

[39] National Academies of Sciences E (2022). *Guidance on PFAS Exposure, Testing, and Clinical Follow-Up*: US.

[40] Buuren S van (2018).

[41] R: the R Project for Statistical Computing. https://www.r-project.org/.

[42] Harlacher E., Wollenhaupt J., Baaten C.C.F.M.J., Noels H. (2022). Impact of uremic toxins on endothelial dysfunction in chronic kidney disease: A systematic review. Int J Mol Sci.

[43] Filipska I., Winiarska A., Knysak M., Stompór T. (2021). Contribution of gut microbiota-derived uremic toxins to the cardiovascular system mineralization. Toxins.

[44] El Chamieh C., Liabeuf S., Massy Z. (2022). Uremic toxins and cardiovascular risk in chronic kidney disease: what have we learned recently beyond the past findings?. Toxins.

[45] Nemet I., Saha P.P., Gupta N. (2020). A cardiovascular disease-linked gut microbial metabolite acts via adrenergic receptors. Cell.

[46] Yang J., Zhou Y., Wang T. (2024). A multi-omics study to monitor senescence-associated secretory phenotypes of Alzheimer’s disease. Ann Clin Transl Neurol.

[47] González-Domínguez R., Rupérez F.J., García-Barrera T., Barbas C., Gómez-Ariza J.L. (2016). Metabolomic-driven elucidation of serum disturbances associated with Alzheimer’s disease and mild cognitive impairment. Curr Alzheimer Res.

[48] Ntranos A., Park H.J., Wentling M. (2022). Bacterial neurotoxic metabolites in multiple sclerosis cerebrospinal fluid and plasma. Brain J Neurol.

[49] Fabresse N., Larabi I.A., Abe E. (2023). Correlation between saliva levels and serum levels of free uremic toxins in healthy volunteers. Toxins.

[50] König M., Palmer K., Malsch C., Steinhagen-Thiessen E., Demuth I. (2024). Polyvascular atherosclerosis and renal dysfunction increase the odds of cognitive impairment in vascular disease: findings of the LipidCardio study. Eur J Med Res.

[51] Levassort H., Boucquemont J., Alencar de Pinho N. (2024). A new approach for cognitive impairment pattern in chronic kidney disease. Nephrol Dial Transplant.

