# Supplementary material for: Association of Phenylacetylglutamine and Cognitive Impairment in CKD
Source: Kidney Int Rep. 2025 May 29;10(8):2720–31. doi: 10.1016/j.ekir.2025.05.037 (PMC12348181; doi:10.1016/j.ekir.2025.05.037)
Supplement: Supplementary File (PDF) — Supplementary Methods 1. List of biological resources centers. Figure S1. (A) PAG and (B) phenylalanine concentrations, by CKD stage (N = 2590). Table S1. Definitions of the operational variables used in the study. Table S2. Association between (A) log2[IS], (B) log2 [TMAO], (C) log2 [pCS], and an MMSE score ≤ 26/30, in unadjusted and adjusted logistic regressions (N = 2590). Table S3. Association between log2[PAG] and an MMSE score < 24/30, in unadjusted and adjusted logistic regressions (N = 2590). Table S4. Association between (A) log2[IS], (B) log2 [TMAO], (C) log2 [pCS], and an MMSE score < 24/30, in unadjusted and adjusted logistic regressions (N = 2590). STROBE checklist. [file mmc1.pdf]

SUPPLEMENTAL MATERIAL

TABLE OF CONTENT:

Supplementary Table S1: Definitions of the operational variables used in the study.

Supplementary Figure S1: PAG (a) and phenylalanine (b) concentrations, by CKD stage (n=2590)

Supplementary Table S2: Association between log2[IS] (a), log2 [TMAO] (b), log2 [pCS] (c), and an MMSE score ≤26/30, in unadjusted and adjusted logistic regressions (n=2590)

Supplementary Table S3: Association between log2[PAG] and an MMSE score <24/30, in unadjusted and adjusted logistic regressions (n=2590)

Supplementary Table S4: Association between log2[IS] (a), log2 [TMAO] (b), log2 [pCS] (c), and an MMSE score <24/30, in unadjusted and adjusted logistic regressions (n=2590)

Supplementary Methods 1: List of biological resources centers

Supplementary Table S5: STROBE checklist

Supplementary Table S1: Definitions of the operational variables used in the study.

Variables	Definitions
Educational level	Educational level was assessed as the number of years in full-time education; in France, a level of 12 years or more corresponds to high school graduation.
Polymedication	Polymedication was defined as taking at least 5 prescription drugs per day. A specific case report form was used to record drug prescriptions during the three months prior to study inclusion.
Anxiolytics	Anxiolytics were defined as drugs in ATC class N05B
Antidepressants	Antidepressants were defined as drugs in ATC classes N06AB and N06AX
Psychoactive drugs	Psychoactive drugs were defined as antidepressants, anxiolytics, or antipsychotics (ATC class N05A)
Hypertension	Hypertension was defined as a history of hypertension or the use of blood-pressure-lowering medication.
Diabetes mellitus	Diabetes mellitus was defined as a history of diabetes, antidiabetic medication use, a glycosylated hemoglobin level ≥6.5%, a fasting glycemia value ≥7 mmol/L, or a non-fasting glycemia value ≥11 mmol/L.
Dyslipidemia	Dyslipidemia was defined as a history of dyslipidemia or the use of lipid-lowering medication.
Obesity	Obesity was defined as a body mass index ≥30 kg/m^2.
Current smoking	Current smoking was noted when patients smoked at least one cigarette per day.
Cardiovascular disease	Cerebrovascular disease was defined as a history of coronary heart disease, angina pectoris, myocardial infarction, coronary bypass surgery, percutaneous coronary intervention, cardiac arrest, atrial fibrillation, other heart rhythm disorder, an implanted pacemaker, implanted defibrillator, heart failure, pulmonary edema, pericarditis, heart valve disease, heart valve prosthesis, stroke, transient ischemic attack, endarterectomy, brain hemorrhage, peripheral vascular disease, intermittent claudication, arterial bypass/percutaneous intervention for arteritis, renal artery stenosis/renal artery surgery, aortic aneurysm, or surgical treatment of an aortic aneurysm
Heart failure	Heart failure was defined as a history of heart failure or pulmonary oedema.
Cerebrovascular disease	Cerebrovascular disease was defined as a history of stroke, carotid endarterectomy, or brain hemorrhage.
Depressive symptoms	The presence of depressive symptoms was assessed using the short form of the Center for Epidemiologic Studies Depression Scale (CESD-10), a 10-item self-administered questionnaire (range: 0-30 points) in which a higher score indicates a greater burden of depressive symptoms.
Current depression	Current depression was defined as either the use of antidepressant medication or a CESD short form score ≥ 10/30. This cutoff is recognized in several studies as effective for detecting depression (with a higher score indicating more pronounced depressive symptoms)
The estimated Glomerular Filtration Rate (eGFR)	The GFR was estimated using the 2009 Chronic Kidney Disease - Epidemiology Collaboration (CKD-EPI) creatinine equation.
The urine albumin-to-creatinine ratio (uACR)	The urinary albumin-to-creatinine ratio (uACR) was measured in only 30% of the participants, while the protein-to-creatinine ratio (PCR), albumin excretion rate (AER) or protein excretion rate (PER) was measured in the others. We therefore used a categorical variable (referred to as the albumin- or protein-to-creatinine ratio) and patients classified according to the Kidney Disease: Improving Global Outcomes 2012 guideline stages, as follows:

	- A1 (normal): ACR<3 (PCR<15)mg/mmol or AER<30 (PER<150)mg/24 h;<br>- A2 (high): ACR 3–30 (PCR 15– 50) mg/mmol or AER 30–300 (PER 150–500) mg/24 h;<br>- A3 (very high): ACR ≥30 (PCR ≥50) mg/mmol or AER ≥300 (PER ≥500) mg/24 h.
Uremic toxins	At baseline, serum samples were collected, immediately stored at 4 ˚C, and aliquoted within 6 hours, without additional processing. All CKD-REIN samples were stored at −80 ◦C) at the Biobanque de Picardie biological resource center and shipped frozen to Paris for analysis. Both teams were blinded to the outcome and the patients' characteristics. UT fractions were assayed in serum using a previously validated ultra-high-performance liquid chromatography-tandem mass spectrometry (LC-MS/MS) technique, as described previously. In the CKD-REIN cohort, serum UT levels of phenylalanine, phenylacetylglutamine, Trimethylamine-N-oxide (TMAO), p-cresyl sulfate and indoxyl sulfate were assayed. These UTs are protein-bound molecules, and their serum concentrations were reported as the free fraction and the total. To determine total UT concentrations, 50 µL of serum were first precipitated with 340 µL of methanol plus 25 µL of isotolabeled internal standards. After centrifugation for 10 min at 9000× g, the supernatant was evaporated under a nitrogen stream and then reconstituted in 80 µL of water. Free UT concentrations were determined by ultrafiltration; 150 µL of serum was introduced into an ultracentrifugal filter (pore size: 30 kDa) and then centrifuged at 13,300× g for 20 min. Given that p-cresyl sulfate and indoxyl sulfate are mainly bound to albumin (65 kDa, which does not pass through the filter), the residual filtrate contained only the free UT fraction.

Supplementary Figure S1: PAG (a) and phenylalanine (b) concentrations, by CKD stage (n=2590)

a

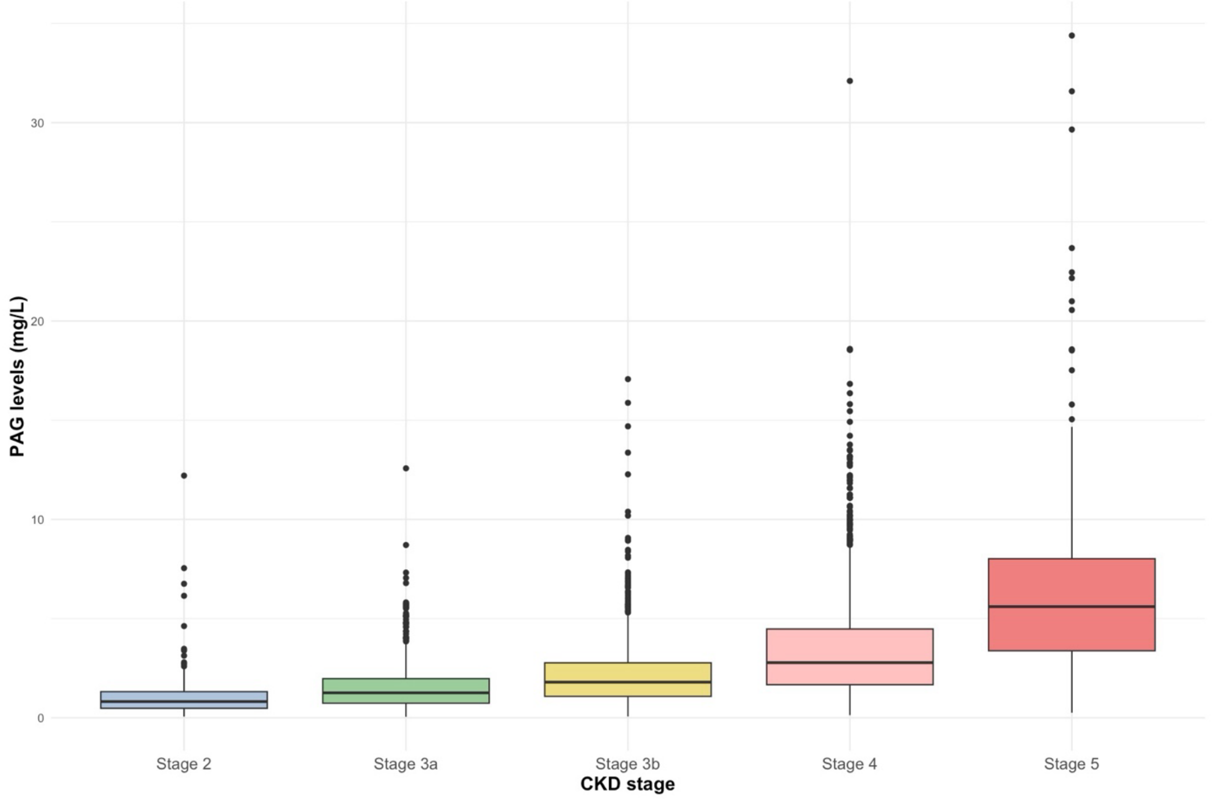

b

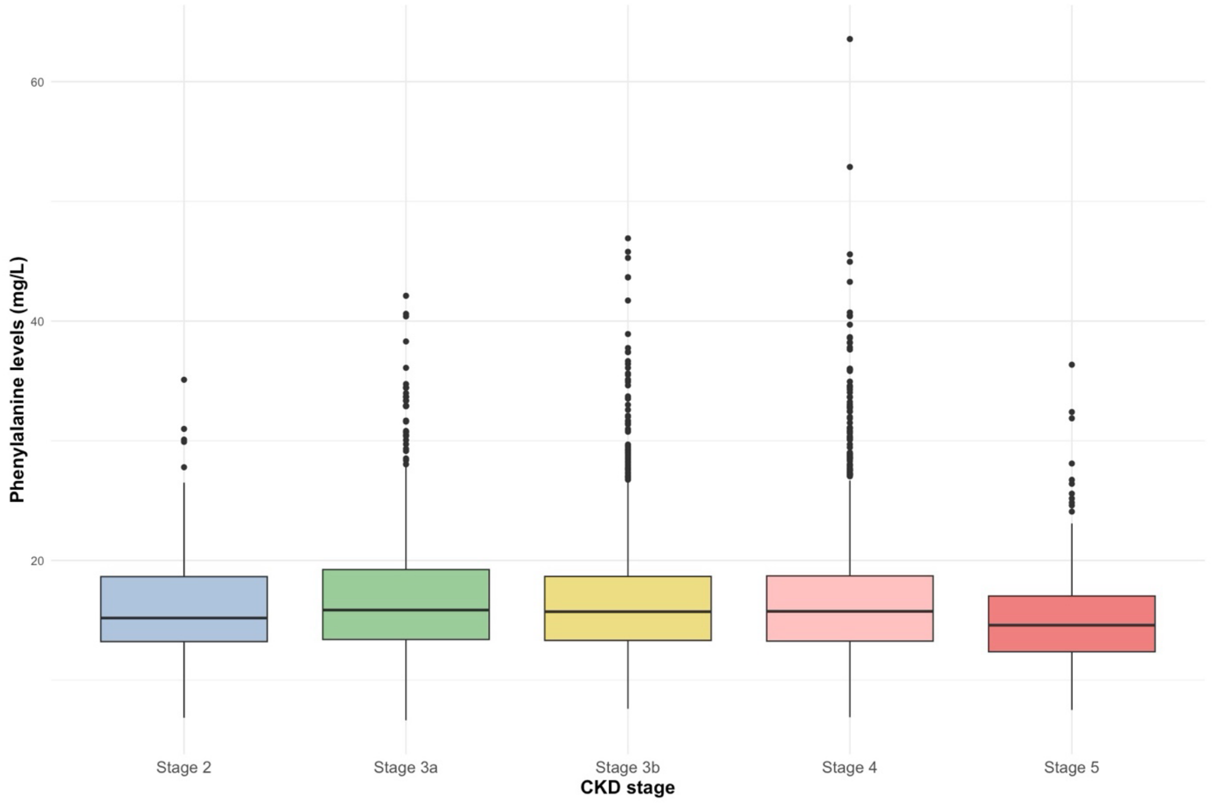

Abbreviations: PAG, phenylacetylglutamine; CKD, chronic kidney disease

Supplementary Table S2: Association between log2[free IS] (a), log2 [total TMAO] (b), log2 [free pCS] (c), and an MMSE score ≤26/30, in unadjusted and adjusted logistic regressions (n=2590)

a: Association between log2[free IS] and an MMSE score ≤26/30, in unadjusted and adjusted logistic regressions (n=2590)

	**Unadjusted**		**Model 1**		**Model 2**		**Model 3**	
	OR [95%CI]	*p*	OR [95%CI]	*p*	OR [95%CI]	*p*	OR [95%CI]	*p*
Uremic toxins								
Log2 [free IS]	1.14 [1.07, 1.21]	**<0.001**	1.11 [1.04, 1.18]	**0.001**	1.10 [1.02, 1.22]	**0.01**	1.10 [1.01, 1.19]	**0.02**

Model 1 was adjusted for Log2 [free IS], age, male sex, and educational level

Model 2 was adjusted for Log2 [free IS], age, male sex, educational level, hypertension, diabetes mellitus, dyslipidemia, obesity, current smoking, cerebrovascular disease, depression, log2Σ(total TMAO, total PAG, free pCS)

Model 3 was adjusted for Log2 [free IS], age, male sex, educational level, hypertension, diabetes mellitus, dyslipidemia, obesity, current smoking, cerebrovascular disease, depression, log2Σ(total TMAO, total PAG, free pCS), eGFR, and uACR

Abbreviations: CI, confidence interval; OR, odds ratio; Log2, based-2 logarithm; MMSE, Mini Mental State Examination; eGFR, estimated glomerular filtration rate; PAG, phenylacetylglutamine; TMAO, trimethylamine oxide; IS, indoxyl sulfate; pCS, p-cresyl sulfate; uACR, urinary albumin-to-creatinine ratio; Σ, sum

b: *Association between log2[total TMAO] and an MMSE score ≤26/30, in unadjusted and adjusted logistic regressions (n=2590)*

	**Unadjusted**		**Model 1**		**Model 2**		**Model 3**	
	OR [95%CI]	*p*	OR [95%CI]	*p*	OR [95%CI]	*p*	OR [95%CI]	*p*
Uremic toxins								
Log2 [total TMAO]	1.01 [0.95, 1.08]	0.6	1.00 [0.94, 1.07]	0.9	0.94 [0.86, 1.02]	0.1	0.93 [0.86, 1.02]	0.1

Model 1 was adjusted for Log2 [total TMAO], age, male sex, and educational level

Model 2 was adjusted for Log2 [total TMAO], age, male sex, educational level, hypertension, diabetes mellitus, dyslipidemia, obesity, current smoking, cerebrovascular disease, depression, log2Σ(free IS, total PAG, free pCS)

Model 3 was adjusted for Log2 [total TMAO], age, male sex, educational level, hypertension, diabetes mellitus, dyslipidemia, obesity, current smoking, cerebrovascular disease, depression, log2Σ(free IS, total PAG, free pCS), eGFR, and uACR

Abbreviations: CI, confidence interval; OR, odds ratio; Log2, based-2 logarithm; MMSE, Mini Mental State Examination; eGFR, estimated glomerular filtration rate; PAG, phenylacetylglutamine; TMAO, trimethylamine oxide; IS, indoxyl sulfate; pCS, p-cresyl sulfate; uACR, urinary albumin-to-creatinine ratio; Σ, sum

c: *Association between log2[free pCS] and an MMSE score ≤26/30, in unadjusted and adjusted logistic regressions (n=2590)*

	**Unadjusted**		**Model 1**		**Model 2**		**Model 3**	
	OR [95%CI]	*p*	OR [95%CI]	*p*	OR [95%CI]	*p*	OR [95%CI]	*p*
Uremic toxins								
Log2 [free pCS]	1.07 [1.02, 1.12]	**0.007**	1.03 [0.98, 1.08]	0.3	0.99 [0.93, 1.06]	0.8	0.99 [0.92, 1.06]	0.8

Model 1 was adjusted for Log2 [free pCS], age, male sex, and educational level

Model 2 was adjusted for Log2 [free pCS], age, male sex, educational level, hypertension, diabetes mellitus, dyslipidemia, obesity, current smoking, cerebrovascular disease, depression, log2Σ(free IS, total PAG, total TMAO)

Model 3 was adjusted for Log2 [free pCS], age, male sex, educational level, hypertension, diabetes mellitus, dyslipidemia, obesity, current smoking, cerebrovascular disease, depression, log2Σ(free IS, total PAG, total TMAO), eGFR, and uACR

Abbreviations: CI, confidence interval; OR, odds ratio; Log2, based-2 logarithm; MMSE, Mini Mental State Examination; eGFR, estimated glomerular filtration rate; PAG, phenylacetylglutamine; TMAO, trimethylamine oxide; IS, indoxyl sulfate; pCS, p-cresyl sulfate; uACR, urinary albumin-to-creatinine ratio; Σ, sum

Supplementary Table S3: Association between log2[PAG] and an MMSE score <24/30, in unadjusted and adjusted logistic regressions (n=2590)

	**Unadjusted**		**Model 1**		**Model 2**		**Model 3**	
	OR [95%CI]	*p*	OR [95%CI]	*p*	OR [95%CI]	*p*	OR [95%CI]	*p*
Uremic toxins								
Log2 [total PAG]	1.19 [1.08,1.31]	**<0.001**	1.10 [0.99,1.22]	0.07	1.07 [0.94,1.22]	0.1	1.05 [0.91,1.20]	0.1

Model 1 was adjusted for Log2 [total PAG], age, male sex, and educational level
Model 2 was adjusted for Log2 [total PAG], age, male sex, educational level, hypertension, diabetes mellitus, dyslipidemia, obesity, current smoking, cerebrovascular disease, depression, log2Σ(total TMAO, free IS, free pCS)
Model 3 was adjusted for Log2 [total PAG], age, male sex, educational level, hypertension, diabetes mellitus, dyslipidemia, obesity, current smoking, cerebrovascular disease, depression, log2Σ(total TMAO, free IS, free pCS), eGFR, and uACR

Abbreviations: CI, confidence interval; OR, odds ratio; Log2, based-2 logarithm; MMSE, Mini Mental State Examination; eGFR, estimated glomerular filtration rate; PAG, phenylacetylglutamine; TMAO, trimethylamine oxide; IS, indoxyl sulfate; pCS, p-cresyl sulfate; uACR, urinary albumin-to-creatinine ratio; Σ, sum

Supplementary Table S4: Association between log2[IS] (a), log2 [TMAO] (b), log2 [pCS] (c), and an MMSE score <24/30, in unadjusted and adjusted logistic regressions (n=2590)

a: Association between log2[free IS] and an MMSE score <24/30, in unadjusted and adjusted logistic regressions (n=2590)

	**Unadjusted**		**Model 1**		**Model 2**		**Model 3**	
	OR [95%CI]	*p*	OR [95%CI]	*p*	OR [95%CI]	*p*	OR [95%CI]	*p*
Uremic toxins								
Log2 [free IS]	1.14 [1.04, 1.24]	**0.003**	1.10 [1.00, 1.20]	0.045	1.05 [0.93, 1.17]	0.4	1.03 [0.91, 1.16]	0.6

Model 1 was adjusted for Log2 [free IS], age, male sex, and educational level

Model 2 was adjusted for Log2 [free IS], age, male sex, educational level, hypertension, diabetes mellitus, dyslipidemia, obesity, current smoking, cerebrovascular disease, depression, log2Σ(total TMAO, total PAG, free pCS)

Model 3 was adjusted for Log2 [free IS], age, male sex, educational level, hypertension, diabetes mellitus, dyslipidemia, obesity, current smoking, cerebrovascular disease, depression, log2Σ(total TMAO, total PAG, free pCS), eGFR, and uACR

Abbreviations: CI, confidence interval; OR, odds ratio; Log2, based-2 logarithm; MMSE, Mini Mental State Examination; eGFR, estimated glomerular filtration rate; PAG, phenylacetylglutamine; TMAO, trimethylamine oxide; IS, indoxyl sulfate; pCS, p-cresyl sulfate; uACR, urinary albumin-to-creatinine ratio; Σ, sum

b: *Association between log2[total TMAO] and an MMSE score <24/30, in unadjusted and adjusted logistic regressions (n=2590)*

	**Unadjusted**		**Model 1**		**Model 2**		**Model 3**	
	OR [95%CI]	*p*	OR [95%CI]	*p*	OR [95%CI]	*p*	OR [95%CI]	*p*
Uremic toxins								
Log2 [total TMAO]	1.07 [0.98, 1.17]	0.1	1.06 [0.96, 1.17]	0.2	1.01 [0.90, 1.14]	0.9	0.97 [0.81, 1.17]	0.7

Model 1 was adjusted for Log2 [total TMAO], age, male sex, and educational level

Model 2 was adjusted for Log2 [total TMAO], age, male sex, educational level, hypertension, diabetes mellitus, dyslipidemia, obesity, current smoking, cerebrovascular disease, depression, log2Σ(free IS, total PAG, free pCS)

Model 3 was adjusted for Log2 [total TMAO], age, male sex, educational level, hypertension, diabetes mellitus, dyslipidemia, obesity, current smoking, cerebrovascular disease, depression, log2Σ(free IS, total PAG, free pCS), eGFR, and uACR

Abbreviations: CI, confidence interval; OR, odds ratio; Log2, based-2 logarithm; MMSE, Mini Mental State Examination; eGFR, estimated glomerular filtration rate; PAG, phenylacetylglutamine; TMAO, trimethylamine oxide; IS, indoxyl sulfate; pCS, p-cresyl sulfate; uACR, urinary albumin-to-creatinine ratio; Σ, sum

c: *Association between log2[free pCS] and an MMSE score <24/30, in unadjusted and adjusted logistic regressions (n=2590)*

	**Unadjusted**		**Model 1**		**Model 2**		**Model 3**	
	OR [95%CI]	*p*	OR [95%CI]	*p*	OR [95%CI]	*p*	OR [95%CI]	*p*
Uremic toxins								
Log2 [free pCS]	1.07 [1.00, 1.15]	0.06	1.01 [0.94, 1.09]	0.7	0.93 [0.85, 1.03]	0.2	0.93 [0.84, 1.02]	0.1

Model 1 was adjusted for Log2 [free pCS], age, male sex, and educational level

Model 2 was adjusted for Log2 [free pCS], age, male sex, educational level, hypertension, diabetes mellitus, dyslipidemia, obesity, current smoking, cerebrovascular disease, depression, log2Σ(free IS, total PAG, total TMAO)

Model 3 was adjusted for Log2 [free pCS], age, male sex, educational level, hypertension, diabetes mellitus, dyslipidemia, obesity, current smoking, cerebrovascular disease, depression, log2Σ(free IS, total PAG, total TMAO), eGFR, and uACR

Abbreviations: CI, confidence interval; OR, odds ratio; Log2, based-2 logarithm; MMSE, Mini Mental State Examination; eGFR, estimated glomerular filtration rate; PAG, phenylacetylglutamine; TMAO, trimethylamine oxide; IS, indoxyl sulfate; pCS, p-cresyl sulfate; uACR, urinary albumin-to-creatinine ratio; Σ, sum

Supplementary Methods 1: List of biological resources centers

The authors would like to thank the teams of all the biological resources centers that participated in the CKD-REIN project:

- Biobanque de Picardie, CRB du Centre Hospitalier Universitaire Amiens Picardie, 1 Rond-Point du Pr Christian Cabrol, 80054 Amiens Cedex 1 (BRIF number: BB-0033-00017)
- NeuroBioTec, CRB des Hospices Civils de Lyon Groupement Hospitalier Est, Hôpital Neurologique, 59 Boulevard Pinel, 69677 Bron Cedex (BRIF number: BB-0033-00046)
- Centre de ressources biologiques du Centre Hospitalier Universitaire de Nantes Hôtel Dieu, Institut de biologie, 9, quai Moncousu, 44093 Nantes Cedex 1 (BRIF number: BB-0033-00040)
- Centre de ressources biologiques du Centre Hospitalier Universitaire Grenoble Alpes, Boulevard de la Chantourne, CS 10217, 38700 La Tronche (BRIF number: BB-0033-00069)
- Centre de ressources biologiques du Centre Hospitalier Régional Universitaire de Nancy, Hôpitaux de Brabois, Bâtiment Recherche Rue du Morvan, 54500 Vandoeuvre-les-Nancy (BRIF number: BB-0033-00035)
- Service de Néphrologie, Centre Hospitalier de Perpignan, 20 Avenue du Languedoc, 66046 Perpignan Cedex 9
- Plateforme de Ressources Biologiques, Hôpital Henri Mondor, 51 avenue du Maréchal de Lattre de Tassigny, 94000 Créteil (BRIF number: BB-0033-00021)
- CIC-1435, Centre d'Investigation Clinique Plurithématique, Centre Hospitalier Universitaire de Limoges, 2 Avenue Martin Luther King, 87042 Limoges Cedex
- Plateforme de Ressources Biologiques de l'Hôpital européen Georges-Pompidou, 20-40 rue Leblanc, 75015 Paris (BRIF number: BB-0033-00063)
- Etablissement Français du sang Hauts de France – Normandie, Site de Bois-Guillaume 609, chemin de la Bretèque, 76235 Bois-Guillaume
- Etablissement Français du sang Nouvelle Aquitaine, site Pellegrin, Place Amélie Raba Léon, CS 21010, 33075 Bordeaux Cedex
- Etablissement Français du sang Hauts de France – Normandie, Site de Loos-Eurasanté, Avenue Pierre Mauroy, Parc Eurasante Epi-de Soil, 59120 Loos
- Etablissement Français du sang Ile de France, Site Avicenne, Hopital Avicenne porte 8, 125 route de Stalingrad, 93009 Bobigny
- Etablissement Français du sang Occitanie, Site de Toulouse, 75 rue de Lisieux, 31300 Toulouse
- Etablissement Français du sang Grand-Est, Site de Colmar, 6 rue du Hohnack, 68025 Colmar Cedex
- Etablissement Français du sang Grand-Est, Site de Metz, 6 rue des Dames de Metz, 57000 Metz
- Etablissement Français du sang PACA-Corse, Site de Marseille, 149, boulevard Baille, 13392 Marseille Cedex 05

Supplementary Table S5: STROBE checklist

	Item No	Recommendation	Page No
**Title and abstract**	1	(*a*) Indicate the study's design with a commonly used term in the title or the abstract	3
		(*b*) Provide in the abstract an informative and balanced summary of what was done and what was found	3
**Introduction**			
Background/rationale	2	Explain the scientific background and rationale for the investigation being reported	4-5
Objectives	3	State specific objectives, including any prespecified hypotheses	5
**Methods**			
Study design	4	Present key elements of study design early in the paper	6
Setting	5	Describe the setting, locations, and relevant dates, including periods of recruitment, exposure, follow-up, and data collection	6
Participants	6	(*a*) Give the eligibility criteria, and the sources and methods of selection of participants. Describe methods of follow-up	6
		(*b*) For matched studies, give matching criteria and number of exposed and unexposed	-
Variables	7	Clearly define all outcomes, exposures, predictors, potential confounders, and effect modifiers. Give diagnostic criteria, if applicable	6-7-8
Data sources/ measurement	8*	For each variable of interest, give sources of data and details of methods of assessment (measurement). Describe comparability of assessment methods if there is more than one group	Supplementary Table S1
Bias	9	Describe any efforts to address potential sources of bias	8-9
Study size	10	Explain how the study size was arrived at	6, Figure 1
Quantitative variables	11	Explain how quantitative variables were handled in the analyses. If applicable, describe which groupings were chosen and why	8-9
Statistical methods	12	(*a*) Describe all statistical methods, including those used to control for confounding	8-9
		(*b*) Describe any methods used to examine subgroups and interactions	8-9-10
		(*c*) Explain how missing data were addressed	9-10
		(*d*) If applicable, explain how loss to follow-up was addressed	-
		(*e*) Describe any sensitivity analyses	9-10
**Results**			
Participants	13*	(a) Report numbers of individuals at each stage of study—eg numbers potentially eligible, examined for eligibility, confirmed eligible, included in the study, completing follow-up, and analysed	11<br>Figure 1
		(b) Give reasons for non-participation at each stage	11
		(c) Consider use of a flow diagram	Figure 1
Descriptive data	14*	(a) Give characteristics of study participants (eg demographic, clinical, social) and information on exposures and potential confounders	11, Table 1
		(b) Indicate number of participants with missing data for each variable of interest	Table 1
		(c) Summarise follow-up time (eg, average and total amount)	-
Outcome data	15*	Report numbers of outcome events or summary measures over time	11

Main results	16	(*a*) Give unadjusted estimates and, if applicable, confounder-adjusted estimates and their precision (eg, 95% confidence interval). Make clear which confounders were adjusted for and why they were included	11-12<br>Table 2
		(*b*) Report category boundaries when continuous variables were categorized	12, Table 3
		(*c*) If relevant, consider translating estimates of relative risk into absolute risk for a meaningful time period	-
Other analyses	17	Report other analyses done—eg analyses of subgroups and interactions, and sensitivity analyses	Table 3, Supplementary Table S2, S3, S4
**Discussion**			
Key results	18	Summarise key results with reference to study objectives	13
Limitations	19	Discuss limitations of the study, taking into account sources of potential bias or imprecision. Discuss both direction and magnitude of any potential bias	15-16
Interpretation	20	Give a cautious overall interpretation of results considering objectives, limitations, multiplicity of analyses, results from similar studies, and other relevant evidence	13-14-15
Generalisability	21	Discuss the generalisability (external validity) of the study results	13-14-15
**Other information**			
Funding	22	Give the source of funding and the role of the funders for the present study and, if applicable, for the original study on which the present article is based	18-19

*Give information separately for exposed and unexposed groups.

Note: An Explanation and Elaboration article discusses each checklist item and gives methodological background and published examples of transparent reporting. The STROBE checklist is best used in conjunction with this article (freely available on the Web sites of PLoS Medicine at http://www.plosmedicine.org/, Annals of Internal Medicine at http://www.annals.org/, and Epidemiology at http://www.epidem.com/). Information on the STROBE Initiative is available at http://www.strobe-statement.org.
